# Temporal stability of spatial cytotype structure in mixed-ploidy populations of *Centaurea stoebe*

**DOI:** 10.1093/aobpla/plac052

**Published:** 2022-10-22

**Authors:** Patrik Mráz, Stanislav Španiel, Katarína Skokanová, Barbora Šingliarová

**Affiliations:** Herbarium Collections & Department of Botany, Faculty of Sciences, Charles University, Benátská 2, CZ-128 01 Prague, Czechia; Plant Science and Biodiversity Centre, Slovak Academy of Sciences, Dúbravská cesta 9, SK-845 23 Bratislava, Slovakia; Plant Science and Biodiversity Centre, Slovak Academy of Sciences, Dúbravská cesta 9, SK-845 23 Bratislava, Slovakia; Plant Science and Biodiversity Centre, Slovak Academy of Sciences, Dúbravská cesta 9, SK-845 23 Bratislava, Slovakia

**Keywords:** *Centaurea stoebe*, colonization, cytotype coexistence, disturbance, flow cytometry, invasion, mixed-ploidy population, polycarpy, polyploidy, secondary contact zone, spatial cytotype structure, spatial segregation

## Abstract

Spatial segregation of cytotypes reduces the negative effect of frequency-dependent mating on the fitness of minority cytotype(s) and thus allows its establishment and coexistence with the majority cytotype in mixed-ploidy populations. Despite its evolutionary importance, the stability of spatial segregation is largely unknown. Furthermore, closely related sympatric cytotypes that differ in their life histories might exhibit contrasting spatial dynamics over time. We studied the temporal stability of spatial structure at a secondary contact zone of co-occurring monocarpic diploids and polycarpic tetraploids of *Centaurea stoebe*, whose tetraploid cytotype has undergone a rapid range expansion in Europe and became invasive in North America. Eleven years after the initial screening, we re-assessed the microspatial distribution of diploids and tetraploids and their affinities to varying vegetation-cover density in three mixed-ploidy populations in Central Europe. We found that overall, spatial patterns and frequencies of both cytotypes in all sites were very similar over time, with one exception. At one site, in one previously purely 2*x* patch, diploids completely disappeared due to intensive succession by shrubby vegetation. The remaining spatial patterns, however, showed the same cytotype clumping and higher frequency of 2*x* despite subtle changes in vegetation-cover densities. In contrast to the expected expansion of polycarpic tetraploids having higher colonization ability when compared to diploids, the tetraploids remained confined to their former microsites and showed no spatial expansion. Spatial patterns of coexisting diploids and tetraploids, which exhibit contrasting life histories, did not change over more than a decade. Such temporal stability is likely caused by relatively stable habitat conditions and very limited seed dispersal. Our results thus imply that in the absence of a disturbance regime connected with frequent human- or animal-mediated seed dispersal, spatial patterns may be very stable over time, thus contributing to the long-term coexistence of cytotypes.

## Teaser

Eleven years after the initial screening, we re-assessed the microspatial distribution of co-occurring monocarpic diploids and polycarpic tetraploids of *Centaurea stoebe*and their affinities to varying vegetation-cover density in three mixed-ploidy populations in Central Europe. Contrary to the expected expansion of polycarpic tetraploids having higher colonization ability when compared to diploids, the tetraploids remained confined to their former microsites and showed no spatial expansion. Our results imply that in the absence of a disturbance regime connected with frequent human- or animal-mediated seed dispersal, spatial patterns may be very stable over time, thus contributing to the long-term coexistence of cytotypes.

## Introduction

Polyploidy, a state in which an organism has more than two haploid sets of chromosomes, is an important evolutionary phenomenon in angiosperms (Comai 2005; Ren *et al.* 2018). By acquiring additional copies of genes across the whole genome, a newly formed polyploid can exhibit new phenotypic and physiological traits with presumably altered adaptive functions (Van der Peer *et al.* 2017; Bomblies 2020). These evolutionary novelties can be efficiently maintained by instantaneous reproductive isolation operating between diploid/lower-ploid ancestor and its neopolyploid progeny, as inter-cytotype crosses often produced non-viable seeds due to malfunction of endosperm (‘triploid block’, Marks 1966; Bretagnole and Thompson 1995; Köhler *et al.* 2010). However, the advantage of a very efficient inter-cytotype reproductive barrier is, at the same time, a disadvantage because rare neopolyploids arising from the majority lower-ploid ancestor have reduced fitness being exposed to much more frequent inter-cytotype crosses. This frequency-dependent fitness disadvantage known as ‘minority cytotype exclusion’ (Levin 1975) operates not only in primary contact zones where neopolyploids are directly formed through the fusion of reduced and unreduced gametes (Ramsey and Schemske 1998) but also in secondary contact zones where two previously geographically separated cytotypes became sympatric. In both situations, the mechanisms preventing inter-cytotype interactions are crucial for the successful establishment of a rare cytotype and its further coexistence (e.g. Kolář *et al.* 2017).

Assortative mating can be assured by several intrinsic and extrinsic mechanisms. The new traits specific to newly formed polyploid in a primary contact zone or polyploid newcomer to the established population of different cytotype(s) in a secondary contact zone might arise from the direct effect of polyploidization *per se* (autopolyploidization) or polyploidization associated with hybridization (allopolyploidization). Such traits might encompass a shift in reproductive strategies like apomixis (Kao 2007) or autogamy (Barringer 2007; Robertson *et al.* 2011), increased clonal growth (Gustafsson 1948; Obeso 2002; Fialová *et al.* 2014). Furthermore, enhanced within-cytotype mating can be achieved by between-cytotype differences related to phenology (non-overlapping flowering, Petit *et al.* 1997; Husband and Sabara 2004; Jersáková *et al.* 2010), pollen competition (Husband *et al.* 2002), pollinator (Segraves and Thompson 1999; Kennedy *et al.* 2006) or ecological preferences (Hülber *et al.* 2009, 2015). From extrinsic mechanisms, the spatial segregation of cytotypes seems to be the most important, as documented in the majority of contact zones studied so far (Kolář *et al.* 2017).

Spatial segregation of cytotypes in contact zones can vary from complete separation (allopatry, e.g. Šafářová and Duchoslav 2010; Castro *et al.* 2012), through parapatric distribution, where cytotypes are intermingled in a very narrow part of a mixed-ploidy site (Baack 2004; Kolář *et al.* 2009; Castro *et al.* 2012), to probably the most often observed mosaic-like pattern with allopatric and sympatric patches variously arranged across a contact zone (e.g. Šafářová and Duchoslav 2010; Trávníček *et al.* 2011a). Importantly, these patterns are scale-dependent; the coexistence of cytotypes is likely over larger spatial scales, while at shorter distances ploidy-uniform clusters prevailed (Trávníček *et al.* 2011a; Šingliarová *et al.* 2019). Non-random cytotype distribution over small spatial scales can be linked to strong cytotype exclusion, clonal reproduction (Chrtek *et al.* 2017; Duchoslav *et al.* 2020), different microhabitat preferences (Fowler and Levin 1984; Sonnleitner *et al.* 2010) or non-adaptive processes, like a founder event with subsequent seed dispersal limitation (Baack 2005; Mráz *et al.* 2012b).

Despite frequently reported spatial segregation patterns in mixed-ploidy species, little is known about whether microspatial cytotypes’ patterns are stable over time or they represent only short-term ‘snapshot’ as suggested by Levin (1975). Over a very large geographical scale (Czechia and Slovakia), a very stable cytogeographic pattern of diploid–tetraploid perennial herb *Viccia craca* was recorded after more than four decades (Trávníček *et al.* 2010). Concerning microspatial distribution of coexisting cytotypes, to our best knowledge, there is also only one study focusing on temporal changes. Čertner *et al.* (2017) studied the co-occurrence of 2*x* and 4*x* of *Tripleurospermum inodorum* (Asteraceae) in 36 mixed-ploidy plots in Czechia. They found that after 1–5 years following the initial screening, a majority of the surveyed plots remained mixed-ploidy. At the same time, however, they recorded considerable fluctuation in cytotypes’ frequencies, and in one plot, which was analysed for spatial cytotype distribution, also profound changes in microspatial cytotype patterns (Čertner *et al.* 2017). High microspatial distribution dynamics in this weed species could be associated with its annual life cycle, as annuals are prone to higher demographic fluctuations when compared to perennial plants (Fone 1989). Furthermore, as annuals/short-lived perennials are frequently growing on disturbed sites with low competition and thus higher chances for successful seedling establishment, their occurrence would rely more on the disturbance regime, which in turn might contribute to the larger spatial and temporal stochasticity when compared to perennials growing under more stable conditions (Janovský and Herben 2020).

Given the extreme scarcity of studies focusing on temporal changes of microspatial cytotype patterns in general which could be, moreover, affected by differences in life history traits, we have decided to re-assess microspatial distribution patterns of *Centaurea stoebe* (spotted knapweed, Asteraceae) in three mixed-ploidy sites assessed in 2009 (Mráz *et al.* 2012b). This herbaceous species consists of two cytotypes, diploid (2*n* = 2*x* = 18) and tetraploid (2*n* = 4*x* = 36), which differ in their life cycle (Ochsmann 2000). While diploids are monocarpic annuals or biannuals, tetraploids are polycarpic perennials (Boggs and Story 1987; Müller 1989; Henery *et al.* 2010; Mráz *et al.* 2011; Hahn *et al.* 2012a). The such between-cytotype difference in the life cycle might provide perennial tetraploids producing seeds repeatedly on the same plant with increased colonization ability when compared to monocarpic diploids (Broz *et al.* 2009). Indeed, the tetraploid cytotype became invasive in its introduced range in North America (Sheley *et al.* 1998; Treier *et al.* 2009; Broennimann *et al.* 2014). Similarly, observations from the native range indicate a recent expansion of 4*x* in Central and Western Europe (Ochsmann 2000; Mráz *et al.* 2012b; Otisková *et al.* 2014), where at certain places tetraploids even replaced diploid plants (Wells *et al.* 2008). The establishment superiority of 4*x* could be achieved, in addition to the perennial life cycle, also by increased inter-cytotype competition which varies along a longitudinal gradient, being more intense in Western Europe (Collins *et al.* 2011). Furthermore, an increased ploidy level is tightly associated with a higher level of heterozygosity in the 4*x* cytotype (Rosche *et al.* 2016). This in turn can reduce inbreeding depression in tetraploids to a larger extent when compared to diploids (Rosche *et al.* 2017), and at the same time contribute to a higher level of plasticity observed in 4*x* cytotype (Hahn *et al.* 2012a, b). These properties make the species a suitable model for assessing spatial dynamics of both cytotypes in their contact zone over time. More specifically, by comparing microscale spatial patterns of 2*x* and 4*x* plants in three mixed-ploidy sites over 11 years, we aim to answer the following fundamental questions: (i) How stable is microspatial distribution of both cytotypes over time? (ii) Have tetraploids colonized new microsites within mixed-ploidy sites?

## Materials and Methods

### Study species

The native range of *C. stoebe* spreads from westernmost Asia to Western Europe with the highest densities recorded in South-Eastern and Eastern Europe (Meusel and Jäger 1992; Ochsmann 2000). The majority of karyologically/cytometrically analysed populations are ploidy-uniform, being either diploid or tetraploid (Ochsmann 2000; Španiel *et al.* 2008; Treier *et al.* 2009; Mráz *et al.* 2012b; Otisková *et al.* 2014). A few mixed-ploidy populations were found mostly in Central Europe, with the highest incidence around Bratislava, Slovakia (Mráz *et al.* 2012b). Microspatial and molecular analyses revealed that these mixed-ploidy sites represent secondary contact zones that were created by the immigration of tetraploids into numerous already established diploid populations. This immigration was favoured by human activities (e.g. quarries for exploitation of sand, stone or gravel; roadsides) which have created new open niches and introduced tetraploid propagules (Mráz *et al.* 2012b). The two cytotypes being morphologically (Ochsmann 2000; Mráz *et al.* 2011) and genetically distinct, the 4*x* is an allotetraploid derivate of the diploid cytotype and another unknown closely related taxon (Mráz *et al.* 2012a), they are treated as separate taxa (usually as subspecies, Ochsmann 2000), but their nomenclature is unresolved (Mráz *et al.* 2011). Both cytotypes are strictly self-incompatible and are insect-pollinated (Harrod and Taylor 1995; Beil *et al.* 2008; P. Mráz *et al.*, unpubl. data). The seeds having very short pappus are dispersed at short distances (<1 m) from mother plants by falling from mature capitula or by flicking of the loosely held achenes due to movement of the stem by wind or passing animals (Sheldon and Burrows 1973; Witztum *et al.* 1996; Colas *et al.* 1997). For longer distances (>1 m), *Centaurea* seeds may be dispersed actively by ants (Imbert 2006; P. Mráz, pers. observ.), grazing animals (attached to their furrows or hooves, Wessels-de Wit and Schwabe 2010) or anthropogenic activities (e.g. attached to wheels of vehicles or to shoes, Sheley *et al.* 1998). The absolute majority of diploids are annual or biannual monocarpic plants, i.e. they die after flowering. Tetraploids are short-lived perennials. Based on herbochronological estimation of several introduced tetraploid populations, the highest frequency of plants was recorded in the age class of 5–9 years (Boggs and Story 1987). Similarly to diploids, tetraploids usually start to flower already in the first year, but then in strong contrast to diploids, they can re-flower for several consecutive years as they are polycarpic (e.g. Boggs and Story 1987; Henery *et al.* 2010; Hahn *et al.* 2012b). Taking into account between-cytotype differences in the size of flower heads, the number of flower heads per plant and life span, tetraploids compared to diploids have much larger seed production over their life (Broz *et al.* 2009).

Despite the generally observed synchrony in flowering between the cytotypes and the observation that pollinators readily move between them in common garden experiments, intermediate triploids (potentially serving as an interploidy bridge for gene flow) are extremely rare (Mráz *et al.* 2012b). This together with the difficulties in obtaining viable triploid progeny from artificial heteroploid crosses (P. Mráz *et al.*, unpubl. data) indicate strong reproductive isolation between the cytotypes and the absence of between-cytotype gene flow.

### Sampling, spatial position and vegetation density assessment

A detailed description, including historical use and management, of sampling sites was published in our previous study (Mráz *et al.* 2012b). Briefly, SAND (Sandberg hill near Devínska Nová Ves, 48.201N, 16.974E) and KOP (Kopáč island on the Danube River near Bratislava, 48.097N, 17.161E) sites represent steppe grasslands where grazing was ceased in the 60s of the last century. Both sites are Nature Reserves. To prevent the succession of shrubs and trees, KOP has been grazed again since 2016 (A. Devečka, pers. comm.), i.e. after our field survey in 2009. The grazing regime at this site involves extensive grazing during 1–2 months with up to 100 animals (sheep and goats), usually in the second half of vegetation season. In addition, in 2019 and 2020 the whole area was mowed once and young trees (mostly *Pinus*) were removed. At locality SAND, only periodic removal of young trees has been carried out. In 2011, when sampling plants and soil for arbuscular mycorrhizis study at SAND site (Sudová *et al.* 2018), we observed that the central part of the area with very dense patches of diploid plants was partially destroyed by very intense trampling of hikers. Therefore, the wooden fence preventing tourists to enter the sandy part of the protected area has been restored and reinforced in 2018. By contrast, TLM (Tlmače village, foothill of Mt. Kusá hora, 48.297N, 18.537E) mixed-ploidy population occurs in a strip of ruderal vegetation along a road and railway. Additionally, numerous diploid plants were also found in 2009 in shrubby and steppe vegetation on a steep slope above the road. Since then, due to very intensive succession, open steppe microsites on this slope have been covered by dense shrubby vegetation. In addition to these three populations, we originally aimed to also re-assess the spatial structure of a mixed population in a former Weit quarry (WEIT site in Mráz *et al.* 2012b). However, since initial screening in 2009, WEIT, specifically its largest grassland part, has been transformed to a small fenced goat farm with a larger goat shelter and *Centaurea* plants completely disappeared.

**Figure 1.**
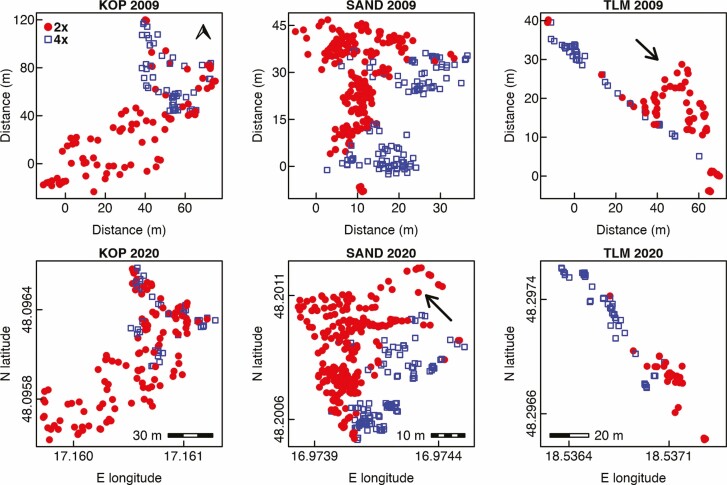
Microspatial distribution of diploid and tetraploid plants of *Centaurea stoebe* in three mixed-ploidy populations surveyed in 2009 and 2020. Note that distances in metres are given for the sampling performed in 2009 when the positions of sampled plants were determined triangularly using a measuring tape, while WGS84 coordinates obtained by ProXH GPS apparatus are given for the same plots visited in 2020. The scale bars inserted in 2020 plots denote the distances along horizontal axes (E longitude). The arrow in the population TLM 2009 shows the patch of 2*x* plants which was not confirmed in the 2020 survey, while the places depicted by an arrow in SAND 2020 were not inspected in 2009.

We assessed the spatial position of cytotypes and collected leaf samples for flow cytometrical analyses from these three mixed-ploidy populations already studied in 2009 (Table 1; Fig. 1; **see****Supporting Information—Fig. S1**). In each selected site, we tried to follow the boundaries of previously sampled areas as precisely as possible, though sometimes the areas were slightly larger than previously (Fig. 1, e.g. SAND site). The plants, irrespective whether of in the flowering or rosette stage, were selected at random but respecting their microscale densities, i.e. more intense sampling was performed in denser patches and vice versa. If possible, we try also to obtain a similar sample size per site as in 2009. However, due to a very efficient spatial position assessment using high-precise GPS apparatus (see below) when compared to the very laborious and, especially on longer distances, also a relatively imprecise triangular method used in the 2009 survey, we sampled more plants at KOP and SAND in the recent survey (Table 1).

**Table 1.** Results of tests and measurements assessing spatial aggregation of diploid and tetraploid plants of *Centaurea stoebe* and vegetation-cover densities of microsites in which these plants were recorded in three mixed-ploidy sites surveyed in the years 2009 and 2020. The numbers of flow cytometrically analysed plants for each of the two cytotypes (2*x*/4*x*) are given in parentheses following the site-year codes. Mantel test—correlation coefficient values (*r*_*m*_). Distance—mean pairwise distance computed separately for diploid and tetraploid plants per site and year with results of spatial aggregation test—SAT (see Materials and Methods). Vegetation density—mean vegetation-cover density of 20 × 20 cm plots around each of 2*x* and 4*x* plants (see Materials and Methods), ↑ denotes a significant increase, while ↓ denotes a significant decrease (Wilcoxon test) in vegetation-cover density per site and ploidy in 2020 when compared to 2009. Vegetation density 2*x* versus 4*x*—signed-rank sum *T* values (Wilcoxon test) corresponding to differences in vegetation-cover densities between 2*x* and 4*x* plants at a given site and year.

Site-year	Mantel test	Distance 2*x *(SAT)	Distance 4*x* (SAT)	Vegetation density 2*x*	Vegetation density 4*x*	Vegetation density 2*x* versus 4*x*
KOP 2009 (83/48)	0.176***	52.3 ± 31.8	29.8 ± 18.8***	2.07 ± 0.75	2.02 ± 0.64	2062.5
KOP 2020 (119/41)	0	60.9 ± 36	36.7 ± 19***	1.86 ± 0.72 (119)↓*	1.93 ± 0.75	2320
SAND 2009 (193/115)	0.263***	17.4 ± 10.8 ***	19.7 ± 11.9***	2.33 ± 1.13	1.82 ± 0.95	13910***
SAND 2020 (274/116)	0.281***	23.6 ± 14.1 ***	20.9 ± 13***	2.32 ± 0.92	2.08 ± 0.89↑*	17294*
TLM 2009 (76/39)	0.430***	23.7 ± 18.3 ***	24.9 ± 20.3**	2.22 ± 0.76	2.05 ± 0.65	1655.5
TLM 2020 (30/43)	0.478***	25.3 ± 22.1 ***	38.3 ± 27.3***	**2.60 ± 1.00↑** ^**MS**^	2.63 ± 1.18↑*	652

values in bold: ^MS^*P* < 0.1 (marginally significant); *P < 0.05; **P < 0.01; ***P < 0.001.

Data on the spatial position of plants were measured using SatLab SLC GNSS receiver (SatLab Geosolutions, Sweden) and collected by MAPUJ application (Geotech Ltd, Slovakia). The averaged measurement accuracy was 1.86 ± 0.23 m at KOP, 0.031 ± 0.005 m at SAND and finally 0.023 ± 0.01 m at TLM site. Because of very high inaccuracy values reported during our field measurements at KOP site, we immediately compared distances between several plants measured by the apparatus and those measured by a manual ruler and found out that these differences were minimal, within 0.01–0.05 m. Unusually high inaccuracy values reported for the KOP site were caused by the inaccessibility of the internet connection on 15 July 2020 from 8 to 12 am, and thus unavailability of RTK (Real-Time Kinematic) data by Slovak real-time positioning service (SKPOS®). Therefore, only accuracy measurements available from the satellite-based positioning system were reported.

Vegetation density on the area of 20 × 20 cm around each spatially assessed plant was estimated using the same approach as in the 2009 survey, i.e. a semi-quantitative scale ranging from 1 (0–5 % vegetation cover) to 5 (75–100 %) and corresponding to the Braun-Blanquet scheme for vegetation relevées (Braun-Blanquet 1928). Details on precise position, altitude, accuracy of measurement, vegetation cover and ploidy level detected for particular plants are in the **Supporting Information—Table S1**.

### Ploidy-level assessment

The samples were analysed using a Partec CyFlow® ML flow cytometer (Partec GmbH, Münster, Germany) equipped with a mercury lamp at the Institute of Botany, Plant Science and Biodiversity Center, Slovak Academy of Sciences in Bratislava and with LED excitation source in Banská Bystrica. Bulk samples (five plants) from fresh leaf tissue were prepared in a two-step procedure using Otto’s extraction buffer and staining buffer containing 4ʹ,6-diamidino-2-phenylindole (DAPI; Doležel *et al.* 1989; Otto 1990). We used leaves of *Lycopersicum esculentum* cv. Stupické polní rané as an internal reference standard (2C = 1.96 pg DNA, Doležel and Bartoš 2005). If a measurement of the bulk sample showed two peaks, plants were re-analysed individually. In four individual plant analyses, we used *Solanum pseudocapsicum* as an internal standard (2C = 2.59 pg DNA, Temsch *et al.* 2010) to avoid overlapping peaks of *L. esculentum* and *Centaurea* samples showing slightly greater genome size than that corresponding to diploid cytotype *C. stoebe* (see Results and **Supporting Information—Table S2**). We inferred the ploidy level of samples as a relative position of the sample G1 peak with respect to that of the internal standard. Fluorescence of at least 5000 particles was recorded; only histograms with symmetrical peaks with a coefficient of variance of the standard and sample G1 peaks below 3.5 % were considered. The ploidy level and the relative DNA content (2C value) were estimated based on the ratio of G1 peak of standard and G1 peak of the sample (the ratio hereinafter referred to as RSS).

### Data analyses

To assess the spatial aggregation of cytotypes within each site we applied three different approaches. First, we performed the Mantel test using the ‘mantel.rtest’ function implemented in the ‘ade4’ package (Thioulouse *et al.* 2018), where the correlation between pairwise geographical distances of plants and pairwise cytotype ‘distances’ (0 for plants of the same cytotype and 1 for different plants) was computed and statistically evaluated using 9999 randomizations. Second, we compared the mean pairwise distance of the plants belonging to the same cytotype and the mean pairwise distances of plants selected at random (Halverson *et al.* 2008) using a Monte Carlo procedure with 9999 permutations. This ‘spatial aggregation test’ (hereafter SAT) assessed using our R script (Šingliarová *et al*. 2019) assumes a significant aggregation of plants belonging to the same cytotype when observed mean pairwise distance between the plants of the same cytotype is shorter than the permuted mean pairwise distance. Third, we computed the Ripley’s *K*-function values (Ripley 1977) using the ‘Kest’ function implemented in the ‘spatstat’ package (Baddeley and Turner 2005). *K*-function, which has been successfully applied in a couple of studies focused on the microspatial cytotype patterns (Trávníček *et al.* 2011a, b), determines the type of spatial patterns, i.e. either regular, independent (= random) or clumped. The *K*-function procedure is based on a comparison of the observed mean density of neighbour plants within a circle of radius *r* of each mapped individual plant within a plot area and the expected density derived from the total number of mapped individuals and the total plot area. Subsequently, the plot based on *K*-function values was transformed to the plot based on *L*-values as recommended to linearize the graphical outputs and minimize the variance (Baddeley and Turner 2005). To assess pairwise spatial inter-cytotype interactions within mixed-ploidy plots, we used the bivariate *K*-function transformed to the bivariate *L*-function indicating either positive, neutral or negative interactions between each individual plant and all neighbour plants belonging to the other cytotype within a circle of radius *r*. Statistical assessment of spatial patterns was performed using a Monte Carlo procedure with 1000 permutations.

Differences in vegetation-cover densities between cytotypes within each site were computed using a non-parametric Wilcoxon test. We used the same approach to assess putative temporal changes (2009 versus 2020) in vegetation-cover densities surrounding the plants of the same ploidy at the same site. All statistical analyses and plotting were performed within the R environment (R Core Team 2020).

## Results

In three mixed-ploidy populations, we sampled 618 plants for which we determined the ploidy level, assessed their spatial position and attributed corresponding vegetation-cover density classes in which these plants occurred (Table 1). The relative position of G1 phase of samples to G1 phase of internal standard ranged from 0.800 to 0.983 in 2*x* samples (mean 0.818 ± 0.024), and from 1.516 to 1.575 (mean 1.539 ± 0.013) in 4*x* samples **[see****Supporting Information—Table S2****]**. Several diploid plants from KOP site had higher values of sample/standard ratio than expected likely due to the presence of either B chromosomes or rare aneuploidy previously recorded within this population (Španiel *et al.* 2008). No triploid plant was recorded.

Overall, we found more diploid than tetraploid plants (423 and 195, respectively) with diploids largely prevailing at KOP and SAND, but not at TLM (Table 1).

**Figure 2.**
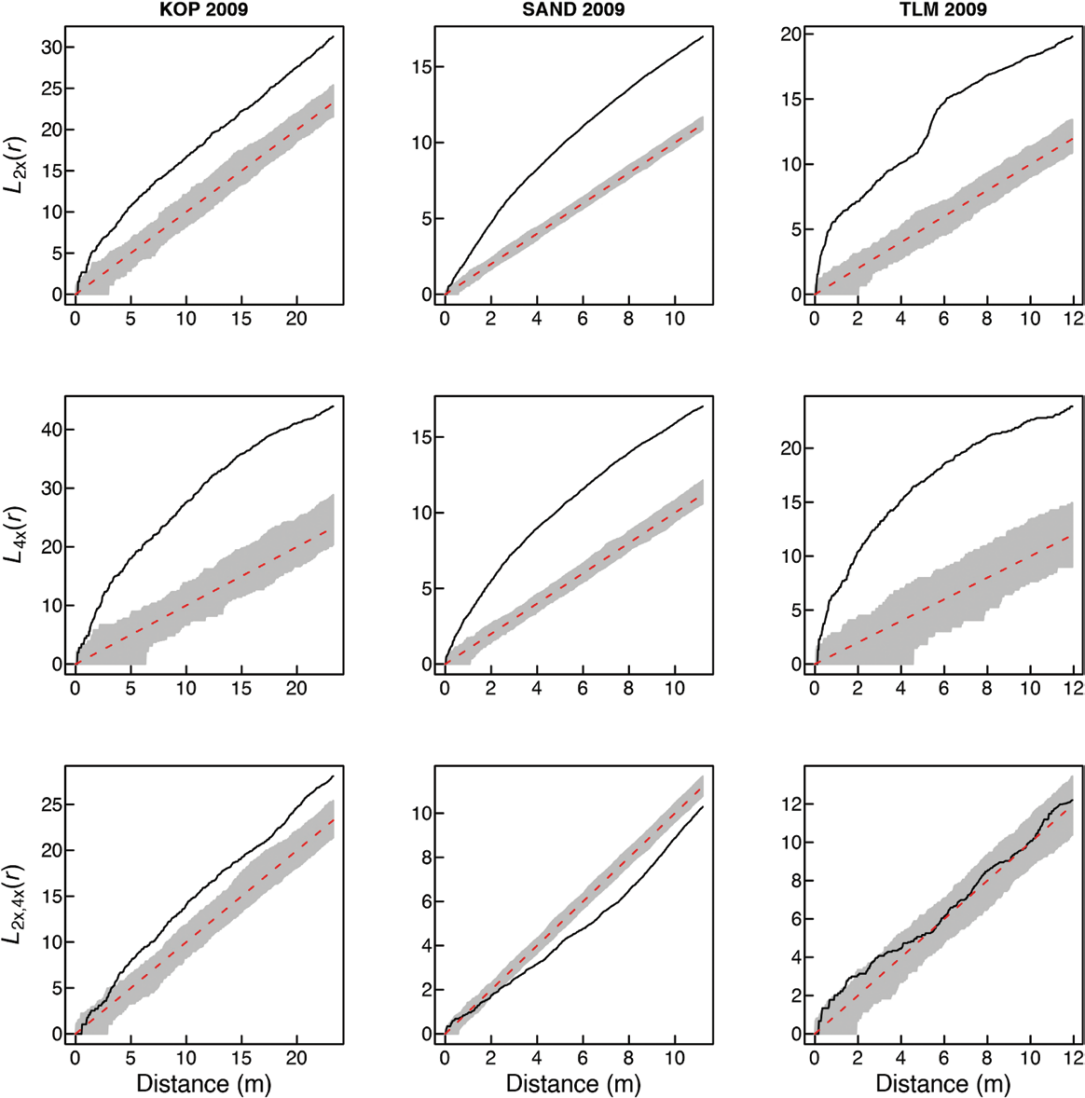
Spatial intra-cytotype aggregation patterns of 2*x* and 4*x* cytotypes of *Centaurea stoebe* (first two rows), and spatial inter-cytotype interaction (the third row) in three mixed-ploidy populations surveyed in 2009. Values of the *L*(*r*)-function are depicted by a thick continuous line, while grey denotes the 95 % confidence intervals. *L*(*r*) values larger than the upper confidence limit in the two first rows indicate significant intra-cytotype aggregations of 2*x* and 4*x* plants at the particular distance of *r*. The third row of plots shows pairwise inter-cytotype associations, where values of the bivariate *L*_2*x*,4*x*_(*r*)-function are shown by a thick continuous line. *L*_2*x*,4*x*_(*r*) values larger than the upper confidence limit indicate a positive inter-cytotype association, values smaller than the lower confidence limit indicate a negative association and finally, *L*_2*x*,4*x*_(*r*) values within confidence intervals indicate a neutral association.

**Figure 3.**
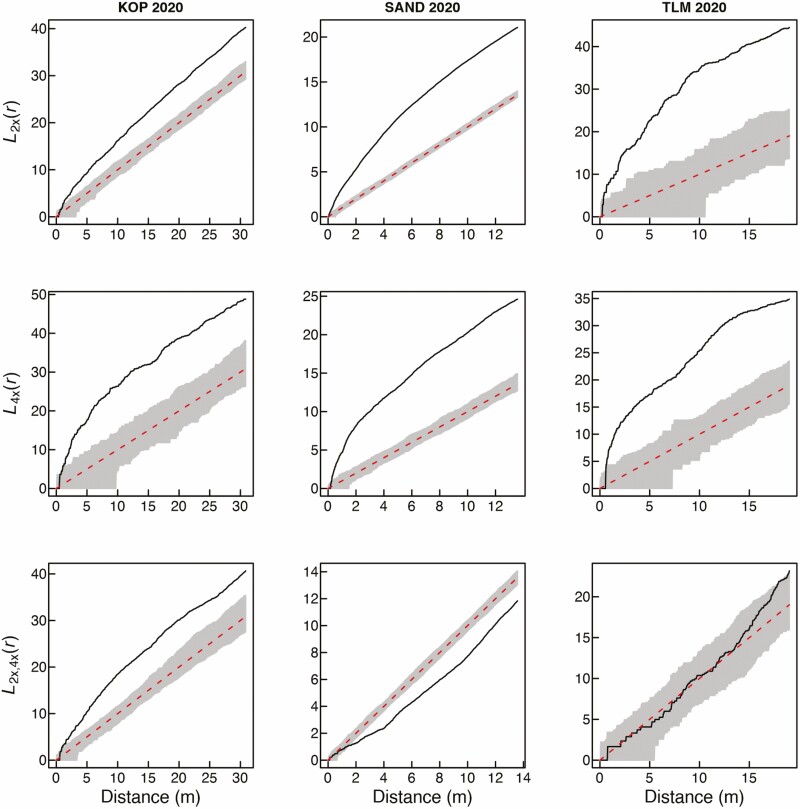
Spatial intra-cytotype aggregation patterns of 2*x* and 4*x* cytotypes of *Centaurea stoebe* (first two rows), and spatial inter-cytotype interactions (the third row) in three mixed-ploidy populations surveyed in 2020. Values of the *L*(*r*)-function are depicted by a thick continuous line, while grey denotes the 95 % confidence intervals. *L*(*r*) values larger than the upper confidence limit in the two first rows indicate significant intra-cytotype aggregations of 2*x* and 4*x* plants at the particular distance of *r*. The third row of plots shows pairwise inter-cytotype associations, where values of the bivariate *L*_2*x*,4*x*_(*r*)-function are shown by a thick continuous line. *L*_2*x*,4*x*_(*r*) values larger than the upper confidence limit indicate a positive inter-cytotype association, values smaller than the lower confidence limit indicate a negative association and finally, *L*_2*x*,4*x*_(*r*) values within confidence intervals indicate a neutral association.

Visual comparison of the overall distribution of diploid and tetraploid plants of *C. stoebe* in three mixed-ploidy populations suggests very high stability of cytotype spatial structure over the period of 11 years (Fig. 1). At KOP site, however, we observed more 2*x* plants intermingled with 4*x* plants in 2020 when compared to 2009. Furthermore, at TLM, one large cluster of diploid plants growing in semi-open steppe in 2009 was represented by only a few individuals in the 2020 survey (Fig. 1), when this part of the site was overgrown by shrubs. Spatial analyses using Mantel test revealed statistically significant aggregation of cytotypes at SAND and TLM, but not at KOP site in 2020 (Table 1). This is likely because in this population, diploids as a major cytotype were very evenly distributed over the whole sampling area (Fig. 1; Table 1—statistically non-significant SAT aggregation) including patches with 4*x* plants. In contrast, the distribution of tetraploids was spatially very limited (Fig. 1; Table 1—statistically significant SAT aggregation). When analysed separately, 2*x* and 4*x* plants showed significantly clumped, i.e. non-random distributions across all three mixed-ploidy plots in both surveyed years (Figs 2 and 3). Inter-cytotype spatial patterns showed either positive (KOP, both years), negative (SAND, both years) and neutral (TLM, both years) associations (Figs 2 and 3), and the *L*-functions showed fairly similar course in between years comparison within each site (Figs 2 and 3).

At KOP and TLM, diploids and tetraploids occurred at microsites with fairly similar vegetation-cover densities (Table 1). In contrast, tetraploids at SAND tended to occupy more open sites when compared to diploids, thus confirming the pattern from 2009 (Table 1). Over the period of 11 years, the average vegetation-cover densities at particular sites, however, changed. Specifically, we recorded an increased vegetation cover around both diploids and tetraploids at TLM site, though in the 2*x* patches this increase was only marginally significant. Similarly, tetraploids at SAND grew in 2020 in more dense vegetation than in 2009. Contrary to these two sites, we observed generally decreased vegetation cover at KOP over time, but only in diploids, this reduction was statistically significant.

## Discussion

Microspatial segregation is a frequently reported mechanism that lowers inter-cytotype gene flow and thus increases the reproductive assurance of a rare cytotype in ploidy-contact zones (Šafářová and Duchoslav 2010; Laport and Ramsey 2015; Garmendia *et al.* 2018). However, microspatial patterns could change over time, especially in polyploid species which encompass cytotypes differing in their competition/persistence abilities like in *C. stoebe*. Recently, the spread of polycarpic 4*x C. stoebe* has been recorded in Europe (Ochsmann 2000; Mráz *et al.* 2012b; Otisková *et al.* 2014), with a replacement of original 2*x* populations at certain places in Germany (Wells *et al.* 2008). We hypothesized, therefore, that the greater colonization ability of polycarpic 4*x* cytotype on one hand, and putatively greater demographic stochasticity of monocarpic 2*x* cytotype on the other, could alter microspatial patterns of cytotypes we recorded at mixed-ploidy sites in 2009 (Mráz *et al.* 2012b). To test this hypothesis, we re-assessed microspatial cytotype distribution in three mixed-ploidy populations in 2020.

The results of this and the previous study (Mráz *et al.* 2012b) show that 2*x* and 4*x* plants can coexist on very small spatial scales (several centimetres). It is in agreement with the experimental results by Collins *et al.* (2011) who studied inter-cytotype competition using plants from several populations (including two mixed-ploidy populations from the present study). They found that although 2*x* plants were more affected by inter-cytotype competition than 4*x* plants, these differences were not significant, thus allowing the close coexistence of both cytotypes. We did not detect any intermediate triploid plants among 623 analysed individuals, which is in agreement with our previous finding that they are extremely rare (two triploid plants from 1307 analysed plants and seedlings, Mráz *et al.* 2012b), confirming the strong reproductive isolation between the cytotypes and absence of between-cytotype gene flow. Overall, spatial patterns of cytotypes at three re-examined mixed-ploidy sites were very similar to those recorded 11 years ago (Figs 1–3). More specifically, conspicuous clumps of tetraploids recorded in 2009 at KOP, SAND and TLM sites have been confirmed at the same microsites in 2020 (Fig. 1). Similarly, at KOP and SAND 2*x* plants clearly prevailed and were present at all microsites sampled in both temporal censuses (Fig. 1). Nevertheless, small irregularities between two surveys have also been found. The most obvious was the absence of numerous 2*x* plants on a steep steppe slope above the road leading to the andesite quarry at TLM site (Fig. 1, the microsite marked by an arrow in TLM 2009 plot). In the 2020 survey, we observed at these places much denser shrubby vegetation (*Crataegus* sp., *Prunus spinosa*, *Rosa* sp.) when compared to the 2009 census. We suppose therefore that monocarpic 2*x* plants could disappear from this part due to strong interspecific competition as *C. stoebe* is a heliophilous species and a very weak competitor. In contrast to TLM site, in several 4*x* patches at KOP site, we recorded more intermingled 2*x* plants in 2020 than in 2009 (Fig. 1). This increase in 2*x* densities could be caused either by an increased dispersal of 2*x* to these microsites, e.g. due the introduction of regular grazing to this steppic locality in 2016 (see Materials and Methods), or the existence of long-term seed bank of 2*x* cytotype at these places. Concerning the latter explanation, Davis *et al.* (1993) found that germinable seeds of *C. stoebe* can persist in the soil at least for 8 years. This means that a long-term soil seed bank can be an important source of new seedlings in natural populations of both monocarpic diploids and short-lived polycarpic tetraploids of *C. stoebe* (Hahn *et al.* 2012a, 2013). The same phenomenon, i.e. the recruitment of 2*x* plants from a seed bank, was likely involved in the re-appearance of a large patch of 2*x* plants situating between the two large 4*x* patches at SAND. At this microsite, being the closest to the hiking path, previously very dense 2*x* plants almost completely disappeared in 2011 because of serious damage by trampling (Appendix S1 in Sudová *et al.* 2018). Diploids reappeared again in 2020 (or earlier) when this microsite was separated from a hiking path by a woody fence (see Materials and Methods) to better protect a large colony of the European bee-eater nesting in the sandy wall bordering this mixed-ploidy site.

The finding that the overall microspatial cytotype patterns were very stable over 11 years is rather surprising given the expected greater demographic stochasticity in monocarpic diploids and better colonization potential of polycarpic tetraploids. In line with our prior expectation, high fluctuations in cytotypes’ frequencies and important gradual reduction of overall population size achieving almost 95 % (from 130 to 7 plants only) over 5 years was recorded in one mixed-ploidy plot of an annual herb *T. inodorum* (Čertner *et al.* 2017). A such strong decrease in population size as in *T. inodorum* we observed in *C. stoebe* only at one steppe microsite at TLM which was likely caused by the strong succession of shrubby vegetation (Fig. 1). Consistently with this observation, at TLM in 2020 we recorded also an increased vegetation cover around 2*x* and 4*x* plants (Table 1). Since in the remaining parts of three mixed-ploidy populations we observe slighter changes in vegetation cover in comparison to this steppe microsite at TLM, we assume that the high temporal stability of microspatial distributional patterns observed in our study sites could be attributed to stable habitat conditions. KOP and SAND mixed-ploidy populations are situated in Nature Reserves representing semi-natural steppe grasslands where vegetation succession is slowed down by unfavourable bedrocks being composed of very porous gravel and sand sediments, but also by the active management of the State Conservation Agency consisting mostly from the occasional removal of shrubs. Furthermore, since 2016 the KOP site has been regularly grazed by a herd of sheep and goats, and mowed in 2019 and 2020, and this intervention likely caused a slight decrease in overall vegetation densities observed in 2020 (Table 1). Trampling by hikers at the margins of a SAND site could further contribute to the maintenance of open sites favourable for *C. stoebe* in this mixed-ploidy population. Paradoxically, however, the movement of tourists and grazing animals did not alter the general microspatial distributional patterns at these two sites, though anthropogenic activities and grazing are important vectors of long-distance dispersal (>1 m) in this species (Sheley *et al.* 1998; Wessels-de Wit and Schwabe 2010). This is especially surprising in respect of polycarpic tetraploids. Despite their superior colonization abilities when compared to monocarpic diploids (Broz *et al.* 2009; Treier *et al.* 2009; Broennimann *et al.* 2014; Rosche *et al.* 2017), they did not establish in the patches previously composed of 2*x* plants (Fig. 1). In our primary study (Mráz *et al.* 2012b), we stated that very intensive anthropogenic activities like sand (at SAND) or gravel exploitations (at KOP), the first lasting for several decades, not only introduced the propagules of 4*x* plants to newly created open microsites, but also contributed to spreads of 4*x* immigrants. These activities, however, ceased completely after these sites became nature reserves in the 1960s to 1970s (Mráz *et al.* 2012b). It seems therefore that despite the presence of current suitable dispersal vectors, the rate of long-distance seed dispersal has probably not been sufficient to change microspatial cytotype patterns over a period of 11 years only.

## Conclusions

In our study, which is to the best of our knowledge, the second one studying the changes in microspatial patterns of coexisting cytotypes over time, we have shown that such microspatial patterns can be temporarily very stable. Temporal stability of cytotype segregation at small spatial scales is therefore another very important aspect involved in the long-term coexistence of cytotypes, as it provides more stable reproductive assurance of spatially isolated cytotypes. We suppose that relatively stable environmental conditions in natural-like sites and limited seed dispersal on one hand, and the absence of current intense anthropogenic disturbances which were likely involved in the origin of these mixed-ploidy populations (Mráz *et al.* 2012b), are the most important underlying factors for such spatial stability over time. Our results, therefore, imply that under such conditions the successful colonization of new microsites by polycarpic tetraploids would take much more time than one decade and that rapid spread of this cytotype observed in the introduced range in North America (Broennimann *et al.* 2014) has to be strongly linked with intense anthropogenic disturbances.

## Supporting Information

The following additional information is available in the online version of this article—

**Table S1.** Information on ploidy level, vegetation cover, exact position and altitude, and positioning accuracy for *Centaurea stoebe* plants analysed in the study.

**Table S2.** Details on flow cytometry measurements of *Centaurea stoebe* plants.

**Figure S1.** Photos of three sites with mixed-ploidy populations of diploid and tetraploid *Centaurea stoebe* surveyed in 2009 and 2020: (A) SAND (Sandberg hill near Devínska Nová Ves, 48.201N, 16.974E); (B) KOP (Kopáč island on the Danube River near Bratislava, 48.097N, 17.161E); (C) TLM (Tlmače village, foothill of Mt. Kusá hora, 48.297N, 18.537E); (D) diploid plant; (E) tetraploid plant.

plac052_suppl_Supplementary_Figure_S1

plac052_suppl_Supplementary_Table_S1

plac052_suppl_Supplementary_Table_S2

## Data Availability

The data are provided as **Supporting Information**.

## Notes

**Evolution & Diversity. Chief Editor**: Jeremy Beaulieu
