# Supplementary material for: Temporal stability of spatial cytotype structure in mixed-ploidy populations of Centaurea stoebe
Source: AoB Plants. 2022 Oct 22;14(6):plac052. doi: 10.1093/aobpla/plac052 (PMC9683110; doi:10.1093/aobpla/plac052)
Supplement: plac052_suppl_Supplementary_Figure_S1 [file plac052_suppl_supplementary_figure_s1.pdf]

Mráz P, Španiel S, Skokanová K, Šingliarová B. 2022. Temporal stability of spatial cytotype structure in mixed-ploidy populations of *Centaurea stoebe*. *AoB PLANTS* 2022: plac052, https://doi.org/10.1093/aobpla/plac052

Supporting Information

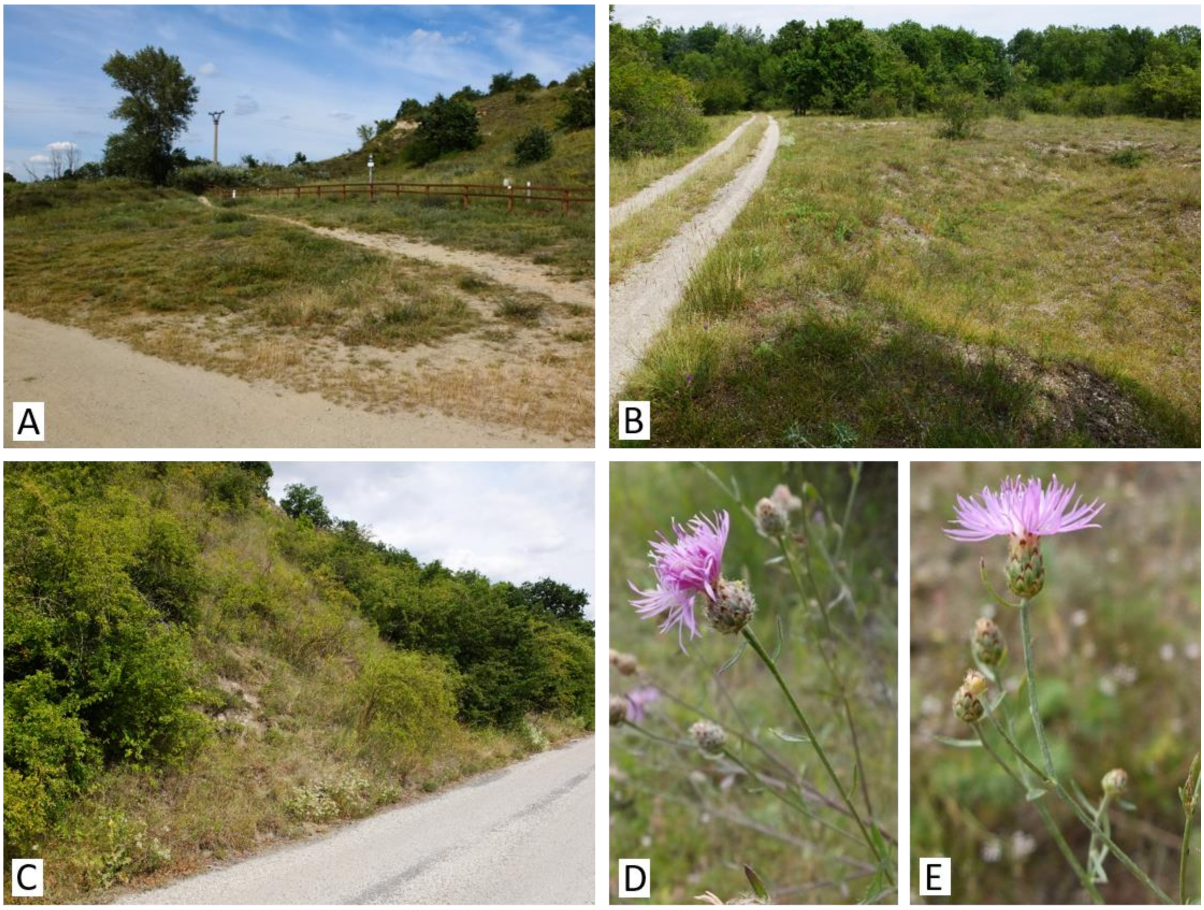

Figure S1: Photos of three sites with mixed-ploidy populations of diploid and tetraploid *Centaurea stoebe* surveyed in 2009 and 2020: (**A**) SAND (Sandberg hill near Devínska Nová Ves, 48.201N, 16.974E); (**B**) KOP (Kopáč island on the Danube River near Bratislava, 48.097N, 17.161E); (**C**) TLM (Tlmače village, foothill of Mt. Kusá hora, 48.297N, 18.537E); (**D**) diploid plant; (**E**) tetraploid plant.
